# Supplementary material for: Group-format, peer-facilitated mental health promotion interventions for students in higher education settings: a scoping review protocol
Source: BMJ Open. 2024 Jun 3;14(6):e080629. doi: 10.1136/bmjopen-2023-080629 (PMC11149131; doi:10.1136/bmjopen-2023-080629)
Supplement: Supplementary data [file bmjopen-2023-080629supp001.pdf]

ID	Author	Publication date	Country	Aims	Population and sample size	Demographics of peers/target population	Methods	Intervention type, comparisons	Peer training	Number of sessions/ Duration of intervention	Outcomes and details of measures	Other comments of relevance for intervention development
