# Supplementary material for: Group-format, peer-facilitated mental health promotion interventions for students in higher education settings: a scoping review protocol
Source: BMJ Open. 2024 Jun 3;14(6):e080629. doi: 10.1136/bmjopen-2023-080629 (PMC11149131; doi:10.1136/bmjopen-2023-080629)
Supplement: Author's manuscript [file bmjopen-2023-080629.draft_revisions.pdf]

BMJ Open is committed to open peer review. As part of this commitment we make the peer review history of every article we publish publicly available.

When an article is published we post the peer reviewers' comments and the authors' responses online. We also post the versions of the paper that were used during peer review. These are the versions that the peer review comments apply to.

The versions of the paper that follow are the versions that were submitted during the peer review process. They are not the versions of record or the final published versions. They should not be cited or distributed as the published version of this manuscript.

BMJ Open is an open access journal and the full, final, typeset and author-corrected version of record of the manuscript is available on our site with no access controls, subscription charges or pay-per-view fees (http://bmjopen.bmj.com).

If you have any questions on BMJ Open's open peer review process please email info.bmjopen@bmj.com

Scoping review protocol for group format, peer-facilitated mental health promotion interventions for students in tertiary education settings

Journal:	*BMJ Open*
Manuscript ID	bmjopen-2023-080629
Article Type:	Protocol
Date Submitted by the Author:	06-Oct-2023

Complete List of Authors:	Brooke-Sumner, Carrie; South African Medical Research Council<br>Machisa, Mercilene T.; South African Med Res Council, Gender and Health Research Unit<br>Sikweyiya, Yandisa; South African Medical Research Council, Gender & Health Research Unit; University of the Witwatersrand, School of Public Health<br>Mahlangu , Pinky; South African Medical Research Council

Keywords:	MENTAL HEALTH, PUBLIC HEALTH, PSYCHIATRY

SCHOLARONE™
Manuscripts

Scoping review protocol for group format, peer-facilitated mental health promotion interventions for students in tertiary education settings

Authors

Carrie Brooke-Sumner[1], Mercy Machisa[2,3], Yandisa Sikweyiya[2,3], Pinky Mahlangu[2,3]

Affiliations

[1]Mental Health, Alcohol, Substance Use and Tobacco Research Unit, South African Medical Research Council, Francie Van Zijl Drive, Parow Valley, Cape Town 7501, South Africa

[2]South African Medical Research Council Gender and Health Research Unit, 1 Soutpansberg Road, Pretoria, Gauteng, South Africa

[3]School of Public Health, Faculty of Health Sciences, University of Witwatersrand, Johannesburg, South Africa, 60 York Road, Parktown, Johannesburg 2193, South Africa.

Corresponding author: carrie.brooke-sumner@mrc.ac.za

Word count: 3479

For peer review only

Abstract

Introduction

Young people in tertiary education face various stressors that can make them vulnerable to mental ill health. Mental health promotion in this group therefore has important potential benefits. Peer-facilitated and group format interventions may be feasible and sustainable for mental health promotion in low- and middle-income countries. The scoping review outlined in this protocol aims to map the literature on group format, peer-facilitated, in-person group interventions for mental health promotion for tertiary education students on campuses in low- and middle-income countries.

Methods and analysis

Relevant studies will be identified through conducting searches of electronic databases, including Medline, CINAHL, Scopus and PsychInfo. Searches will be conducted using Boolean operators (AND, OR, NOT) and truncation functions appropriate for each database Since the relevant academic published literature from LMIC may be limited, we will include a grey literature search. We will include articles from student participants age ranges 18-30 of any gender, and published in peer reviewed journals between 2007 and 2023. We will include English language studies and all study types including RCTs, pilot studies and descriptive studies of intervention development.

For peer review only - http://bmjopen.bmj.com/site/about/guidelines.xhtml

A draft charting table has been developed, which includes the fields: author, publication date, country/countries, aims, population and sample size, methods, intervention type, comparisons, duration of intervention, outcomes and details of measures. The data charting tables will be collated and reviewed by the whole review team.

Ethics and dissemination

No primary data will be collected from research participants to produce this review. All data will be collated from published peer reviewed studies already in the public domain. We will publish the review in an open access, peer reviewed journal accessible to researchers in low-and middle-income countries. This protocol is registered on Open Science Framework (https://osf.io/agbfj/).

Article summary

Strengths and limitations

- This review seeks to establish a body of literature that can inform work of researchers and practitioners in the area of mental health promotion for students in low-and middle income countries.
- The methods are grounded in established methods and guidance for scoping reviews.
- This review will include a search of published and grey literature to synthesize the range of interventions for group based peer-facilitated mental health promotion for students.
- The protocol is limited in focusing on English language articles.

Keywords

Mental health, young adult, college, university, peer, mental health promotion

Background

Mental health of young people is a global public health priority [1], with COVID-19 accentuating the urgency of addressing this area [2, 3]. Mental well-being is crucial for tertiary students to be resilient in facing the demands of academic life [4] as well as being a building block for every society through youth development. Young people entering tertiary education face a period of substantial change, with new and varied stressors and changing interpersonal relationships, financial circumstances, and life challenges [5-7]. Academic and other stress is associated with the development of mental health conditions [5, 7]. Mental distress is prevalent in higher education

For peer review only

For peer review only - http://bmjopen.bmj.com/site/about/guidelines.xhtml

institutions with young women students particularly vulnerable to conditions such as anxiety, depression, and post-traumatic stress disorder (e.g. [8-11]). The stressors experienced in student life overlay other vulnerability factors and social determinants [12] for developing a mental health condition, including previous experiences of childhood adversity and trauma [13, 14], and being from a sexual minority group or gender non-conforming [13]. In low- and middle-income countries (LMIC) in which human resources for mental health treatments are especially scarce [15], the potential gains from prevention of mental disorders for young people are significant since prevention in asymptomatic young people can correspond to population level benefits in the long term [16, 17].

Interventions for promotion of good mental health, and prevention of the development of mental health conditions remain underprioritized in many settings in comparison with efforts for treatment provision, yet such work is a crucial component of the WHO mix of mental health services [18]. Good mental health for young people may be contextually nuanced, but is dependent on a range of domains, including mental health literacy, attitudes towards mental health conditions, self-perceptions, cognitive skills, academic performance, emotions, behaviours, self-management, social skills, quality of significant relationships, physical and sexual health, meaning in life and quality of life [19, 20]. With this range of domains, transdiagnositic or common elements treatment and prevention approaches are relevant, underpinned conceptually by the fostering of resilience through building adaptive social and emotional skills, stress management, positive self-perceptions and building supportive relationships [23, 27, 28. Prevention in relation to mental ill health also encompasses other health behaviours (e.g. dietary health, physical activity, sleep) that can have an impact on mental health outcomes [21]. Harnessing common practice elements can enable an evidence-informed approach to prevention even when a specific evidence-based intervention may be lacking (as may be the case in many LMIC settings where this research area is less developed) [17, 22].

Given the breadth of domains of good mental health for tertiary students, mental health promotion and prevention interventions for young people necessarily cover a wide variety of interventions at the individual, community and societal levels [23] which can be universal (population based) or selective (targeting individuals or subgroups at higher risk) [16, 24]. Young people in higher

For peer review only - http://bmjopen.bmj.com/site/about/guidelines.xhtml

education settings present a particular set of opportunities for preventive and promotive interventions given the social connectedness of student populations. Given that many mental health conditions have their onset in adolescence this is a key point for intervention and there is developing evidence that school-based prevention programmes can be effective in improving mental health literacy and reducing mental health stigma defined as attitudes and beliefs regarding mental disorders [25-28].

There is also a developing systematized evidence base for tertiary student mental health promotion from high income countries (HIC). Systematic reviews of prevention programmes for student mental health showed moderate effects for common practice elements including psychoeducation, relaxation techniques, and cognitive restructuring [19] as well as guided mental health skills training programmes [22, 29-31] and computer and web-based interventions delivered by a variety of professionals [32-34].

Psychoeducation entails mental health literacy training which aims to increase knowledge around mental health conditions (e.g. risk factors, signs and symptoms, knowledge of which may be lower amongst young people than knowledge of physical conditions [18], to reduce stigma and increase help seeking [16]). The evidence suggests that this type of intervention leads to improvement in knowledge around mental health as opposed to help seeking behaviour [17]. Mental health literacy can also improve emotional awareness and knowledge that can be linked to building resilience and improving self-esteem [16]. Current developments in this field include a movement towards a positive conceptualisation of mental health literacy which is not focused on 'illness' and symptoms. Within this conceptualisation, positive mental health literacy includes problem solving ability, independence, relational skills and self-control [38-41]. A similar evidence base for student mental health promotion in LMIC is required [35] given contextual differences in these environments.

Most notably, a key aspect of mental health prevention interventions is the delivery agent. In LMIC settings specialists such as psychologists, social workers and counsellors are scarce [36] and task-sharing (service provision by non-specialists with upward referral if necessary) has long been advocated as a feasible approach for mental health service delivery [23, 37, 38]. Thus there are

For peer review only - http://bmjopen.bmj.com/site/about/guidelines.xhtml

further evidence gaps in relation to tertiary student's mental health promotion in LMICs including the delivery method and agent. Digital interventions have been put forward as a low-resource approach for mental health promotion [35]. Evidence for digital interventions for mental health and access to technology in HIC and LMIC is growing and indicates potential for these approaches, in conjunction with other supportive service options [39, 40]. In-person interventions contribute to a different niche in the potential mix of supportive services, and group approaches may be another feasible low-resource approach in some LMIC contexts. The group format presents the opportunity for reaching higher numbers of students in a less resource intensive approach than individual level support which may be advantageous for mental health promotion [35]. Further to this, peer-led approaches, in which people who share a common lived experience (e.g. being a tertiary student) are involved in providing services, have traction in other areas of public health, for young people in particular (e.g. [41, 42]) and in other areas of mental health service provision (e.g. [43, 44]). Peer facilitated approaches may be particularly appropriate for mental health promotion amongst young people given the importance of peer influence at this point in the life course [45]. These approaches have the potential to be low resource and sustainable in resource constrained settings [41].

This scoping review will map the evidence for in person, group-based, peer-facilitated mental health promotion and prevention interventions for young people in education and training settings in LMIC. Once conducted, this review will enable progress in establishing evidence for such preventive approaches applicable in LMIC. This will serve as a step towards building the field of evidence-informed mental health promotion among students in tertiary education settings in LMIC.

Research questions

1. What range of group based, peer facilitated mental health promotion interventions are offered to tertiary students on LMIC campuses?
2. What are the common practices, processes and outcome measures used for these interventions?

Objectives

1. To map the literature on group format, peer facilitated, in- person group interventions for

For peer review only - http://bmjopen.bmj.com/site/about/guidelines.xhtml

mental health promotion for tertiary education students on campuses in LMIC.

2. To describe these peer-facilitated group interventions and the ways they have been evaluated.
3. To describe the content and intervention delivery (common practice elements) of interventions that have shown positive effects on mental health in these settings.

Methods and analysis

Methodology for the review will be based on that presented by Arksey and O'Malley [46] and further developed by Levac [47]. A key recommendation from this advancement relates to clarifying and linking the purpose of the review with the research question [47]. The purpose of review will be to identify common practice elements of group-format, peer-facilitated mental health promotion interventions used in university/college populations in LMIC. A further recommendation on balancing feasibility with breadth [47] will be implemented through the article search strategy. Since the relevant academic published literature from LMIC may be limited, we will include a grey literature search as recommended by Joanna Briggs Institute Guidance [48, 49]. We will also use an iterative approach for identifying and selecting studies [47] including identifying studies from reference lists of relevant studies with discussion of the review team. The following steps will be undertaken to investigate the review question and are detailed in the sections following:

1. Identifying the research question
2. Identifying relevant studies
3. Study selection
4. Charting the data
5. Collating, summarizing, and reporting results

1.Identifying the research question

The Population Concept Context (PCC) mnemonic has been identified as appropriate for scoping review methodology [48]. The population of interest is defined as students at higher education campuses (aged 18-30) in LMIC; the concept is defined as any mental health promotion intervention delivered in person by a peer and in a group format; context is defined as articles from

For peer review only - http://bmjopen.bmj.com/site/about/guidelines.xhtml

LMIC as defined by World Bank Criteria at the time of the searches. This scoping review therefore have the following research questions:

Primary research question

- What evidence is available globally for peer-facilitated, group-format in-person interventions for mental health promotion for students on higher education campuses in LMIC?

Secondary research questions

- What are the common practice elements and delivery methods for interventions reported in this body of literature?
- What research gaps are identified by this literature included in the scoping review?

2. Identifying relevant studies

Relevant studies will be identified through conducting searches of electronic databases, including Medline, CINAHL, Scopus and PsychInfo. Searches will be conducted using Boolean operators (AND, OR, NOT) and truncation functions appropriate for each database. Search strings will be developed according to the database and will include for example: "mental health literacy OR mental health promotion" AND "intervention" OR "program" OR "mental health intervention" AND "group intervention" OR "peer" AND "student" OR "campus" OR "university" OR "college" (Figure 1). Terms related to positive mental health literacy will also be included (e.g. self care, resilience, autonomy, self-control, self-acceptance, stress management).

We will seek assistance from a specialist librarian in refining search strings for databases based on these key terms. We will take an iterative approach to the searches [48] to include relevant keywords and to maintain feasibility of the number of article records to review. Review authors will also look at reference lists of all articles selected for inclusion as an approach to identify further relevant studies and search for the articles identified from the reference lists. If required, review authors will contact corresponding authors of these articles to identify relevant studies [48]. To further develop the search we will also run searches in Google Scholar and Directory of Open Access Journals based on key words identified in the iterative searching process. The grey literature search will be conducted with terms above using databases such as WorldCat, OpenMD and Eldis. Relevant studies will be downloaded and managed using Endnote Database software to enable removal of duplicates and conducting screening.

For peer review only - http://bmjopen.bmj.com/site/about/guidelines.xhtml

3. Study selection

Titles and abstracts of identified studies will be subjected to double screening for relevance, with records divided among authors to facilitate faster screening. Titles and abstracts will be screened according to the pre-developed inclusion and exclusion criteria developed for answering the review question. Articles identified as relevant in this first screening process will then be included in a full text review conducted by all authors (double screening, two authors review each article). Agreement and disagreement on full text inclusion will be discussed by reviewing authors, and any disagreements resolved through discussion with the full review team.

Inclusion criteria

The purposes of the scoping review mean that included articles will describe in-person interventions delivered on higher education campuses in HIC and LMIC, by peers. We will include articles that describe group-format interventions delivered in college or university settings among student participants age ranges 18-30 of any gender, and published in peer reviewed journals between 2007 and 2023. We will include English language studies and all study types including RCTs, pilot studies and descriptive studies of intervention development.

Exclusion criteria

Articles describing individual and group interventions delivered by clinical professionals (e.g. psychologists, social workers, counsellors or other university staff) will be excluded, as well as articles reporting on clinical treatment interventions as opposed to prevention interventions. Articles reporting on interventions not delivered in person, by peers or in groups (E.g. online/web based) will also be excluded. Non- English language papers and those published outside of the time period indicated will be excluded.

4. Data extraction and management

Data extraction from will be conducted from PDF or other electronic files into an Excel based data management file (Supplementary File 1). We will conduct a pilot test of this abstraction form with initial articles to ensure consistency in data extraction between team members.

5. Charting the data

For peer review only - http://bmjopen.bmj.com/site/about/guidelines.xhtml

The charting process will provide a descriptive summary of the evidence presented in included studies [48], as the initial step towards answering the primary and secondary research questions. A draft charting table has been developed, which includes the fields: author, publication date, country/countries, aims, population and sample size, methods, intervention type, comparisons, duration of intervention, outcomes and details of measures. The data charting tables will be collated and reviewed by the whole review team to address any inconsistencies.

6. Assessment of study quality

For this scoping review we will not conduct a formal assessment of Risk of Bias using one of the established reporting tools (e.g. [50, 51]). We will however use The Template for Intervention Description and Replication (TIDieR) [52] checklist to assess completeness in the reporting of included intervention studies. Available since 2014 this guide may have been used by study authors to describe interventions, and can be used in this scoping review to provide a thorough characterization of interventions.

7. Summarizing and reporting findings

Appropriate tables will be used to give the reader an overall picture of the spread of evidence across publication years and country of origin. A narrative description of the studies will also be presented, aligning findings with the primary and secondary research questions [48]. For ease of use for the reader, findings will be presented under the conceptual headings “Intervention practice elements” and “Intervention delivery” and “Research gaps”. The final manuscript will be prepared using the PRISMA extension for scoping reviews [53]. Depending on the amount of literature found, data abstraction may also include categorizing the extracted information into themes to identify patterns across the literature.

Ethical considerations and Dissemination

No primary data will be collected from research participants to produce this review. All data will be collated from published peer reviewed studies already in the public domain. This study therefore does not require ethical approval from a Human Research Ethics Committee. To minimize potential bias and ensure an accurate reflection of the scope of the literature we have a review team with four members who will work collaboratively. These researchers are senior scientists at the

For peer review only - http://bmjopen.bmj.com/site/about/guidelines.xhtml

South African Medical Research Council, with backgrounds in quantitative and qualitative research in public health and mental health. We will publish the review in an open access, peer reviewed journal accessible to researchers in LMIC. We will also disseminate findings through our established Community Advisory Board and stakeholder engagement platforms of the South African Medical Research Council.

Discussion

The United Nations Sustainable Development Goals (SDGs) prioritise mental wellness in SDG 3 [54]. This indicates high level commitment to improvement in mental wellbeing, which requires a response from all sectors of society, and quality research to guide this response [55]. Addressing social determinants of mental health [12], the social context that precedes the development of mental health conditions, holds great potential for prevention of the growing burden of mental disorders globally and in young people in particular. Young people in tertiary education in particular face developmental and academic stressors which can make them vulnerable to mental health conditions [56-58].

Prevention efforts that promote mental health will need to begin to address vulnerabilities as well as improve coping and resilience. Intervention research to develop and test interventions in this area is required. [55]. The scoping review outlined in this protocol will contribute to moving the mental health promotion field in LMIC forward through mapping group-format, peer-facilitated mental health promotion practice elements. This will add to the body of work on adaptation of complex interventions [59, 60], incorporating learnings from HIC settings with practice elements that are appropriate and can be tailored for LMIC settings. Evidence for approaches that are feasible, acceptable, and crucially - tailored - for LMIC contexts is required, given the risk factors associated with mental health conditions in these settings [57, 61]. For example such approaches may give prominence to developing social support, promoting aspects of wellbeing including social, academic, and spiritual wellbeing, and building resilience and coping strategies which are crucial in LMIC contexts [62]. Through mapping the body of literature this scoping review will also contribute to evidence for state of the field of task-sharing for mental health promotion in LMIC settings. Finally, findings from the review will identify gaps and opportunities for

For peer review only - http://bmjopen.bmj.com/site/about/guidelines.xhtml

researchers and practitioners interested in implementing group-format, peer-led approaches for improving mental health for students in LMIC.

Funding statement

CBS, MM, PM and YS are supported in this work by funds from the South African Medical Research Council.

Author contributions

All authors conceptualized the study and methods collaboratively. CBS produced the initial draft of this manuscript, all authors reviewed and approved the final manuscript.

Competing interests

None declared.

Patient and public involvement

Patients and/or the public were not involved in the design, or conduct, or reporting, or dissemination plans of this research.

Patient consent for publication

Not applicable.

Provenance and peer review

Not commissioned; externally peer reviewed.

Data availability statement

All data for the review will be made available on reasonable request from Kholiswa Dube Kholiswa.Dube@mrc.ac.za or Carrie Brooke-Sumner carrie.brooke-sumner@mrc.ac.za.

Supplemental material

Data charting template.

References

1. Patel, V., et al., *Mental health of young people: a global public-health challenge.* Lancet, 2007. **369**(9569): p. 1302-1313.
2. WHO, *Mental Health and COVID-19: Early evidence of the pandemic's impact*. 2022, World Health Organization: Geneva.
3. Visser, M. and E. Law-van Wyk, *University students' mental health and emotional wellbeing during the COVID-19 pandemic and ensuing lockdown.* South African Journal of Psychology, 2021. **51**(2): p. 229-243.
4. Ahorsu, D.K., et al., *Effect of a peer-led intervention combining mental health promotion with coping-strategy-based workshops on mental health awareness, help-seeking behavior, and*

For peer review only

For peer review only - http://bmjopen.bmj.com/site/about/guidelines.xhtml

wellbeing among university students in Hong Kong. International Journal of Mental Health Systems, 2021. **15**(1): p. 1-10.
5. Karyotaki, E., et al., *Sources of Stress and Their Associations With Mental Disorders Among College Students: Results of the World Health Organization World Mental Health Surveys International College Student Initiative.* Front Psychol, 2020. **11**: p. 1759.
6. Jessop, D.C., C. Herberts, and L. Solomon, *The impact of financial circumstances on student health.* Br J Health Psychol, 2005. **10**(Pt 3): p. 421-39.
7. Liu, X., S. Ping, and W. Gao, *Changes in Undergraduate Students' Psychological Well-Being as They Experience University Life.* Int J Environ Res Public Health, 2019. **16**(16).
8. Macaskill, A., *The mental health of university students in the United Kingdom.* British Journal of Guidance & Counselling, 2013. **41**(4): p. 426-441.
9. Storrie, K., K. Ahern, and A. Tuckett, *A systematic review: Students with mental health problems--a growing problem.* Int J Nurs Pract, 2010. **16**(1): p. 1-6.
10. Rotenstein, L.S., et al., *Prevalence of Depression, Depressive Symptoms, and Suicidal Ideation Among Medical Students: A Systematic Review and Meta-Analysis.* Jama, 2016. **316**(21): p. 2214-2236.
11. Eskin, M., et al., *Suicidal Behavior and Psychological Distress in University Students: A 12-nation Study.* Arch Suicide Res, 2016. **20**(3): p. 369-88.
12. Lund, C., et al., *Social determinants of mental disorders and the Sustainable Development Goals: a systematic review of reviews.* Lancet Psychiatry, 2018. **5**(4): p. 357-369.
13. Campbell, F., et al., *Factors that influence mental health of university and college students in the UK: a systematic review.* BMC Public Health, 2022. **22**(1): p. 1-22.
14. Berenz, E.C., et al., *Posttraumatic stress disorder symptoms, anxiety sensitivity, and alcohol-use motives in college students with a history of interpersonal trauma.* Psychol Addict Behav, 2016. **30**(7): p. 755-763.
15. Rathod, S., et al., *Mental Health Service Provision in Low- and Middle-Income Countries.* Health Serv Insights, 2017. **10**: p. 1178632917694350.
16. Salazar de Pablo, G., et al., *Universal and selective interventions to promote good mental health in young people: Systematic review and meta-analysis.* Eur Neuropsychopharmacol, 2020. **41**: p. 28-39.
17. Colizzi, M., A. Lasalvia, and M. Ruggeri, *Prevention and early intervention in youth mental health: is it time for a multidisciplinary and trans-diagnostic model for care?* International Journal of Mental Health Systems, 2020. **14**(1): p. 23.
18. Shidhaye R, L.C., Chisholm D., *Health Care Platform Interventions. Mental, Neurological, and Substance Use Disorders: Disease Control Priorities*, C.D. Patel V, Dua T, et al., editors., Editor. 2016, The World Bank: Washington (DC).
19. Fusar-Poli, P., et al., *What is good mental health? A scoping review.* Eur Neuropsychopharmacol, 2020. **31**: p. 33-46.
20. Dodd, A.L. and M. Priestley, *University student well-being in the United Kingdom: a scoping review of its conceptualisation and measurement.* 2021. **30**(3): p. 375-387.
21. Hutchesson, M.J., et al., *Health behavior interventions for university students measuring mental health outcomes: A scoping review.* Front Public Health, 2022. **10**: p. 1063429.
22. Rith-Najarian, L.R., M.M. Boustani, and B.F. Chorpita, *A systematic review of prevention programs targeting depression, anxiety, and stress in university students.* J Affect Disord, 2019. **257**: p. 568-584.
23. Petersen, I., et al., *Promotion, prevention and protection: interventions at the population- and community-levels for mental, neurological and substance use disorders in low- and middle-income countries.* Int J Ment Health Syst, 2016. **10**: p. 30.

For peer review only
For peer review only - http://bmjopen.bmj.com/site/about/guidelines.xhtml

24. Organization, W.H., *Prevention of Mental Disorders: Effective Interventions and Policy Options: Summary Report*. 2004: Geneva.
25. Ma, K.K.Y. and J.K. Anderson, *Review: School-based interventions to improve mental health literacy and reduce mental health stigma - a systematic review.* 2023. **28**(2): p. 230-240.
26. Ma, K.K.Y. and A.M. Burn, *Review: School-based mental health literacy interventions to promote help-seeking - a systematic review.* 2022.
27. Amudhan, S., *Project SUMS (scaling up of mental health in schools): design and methods for a pragmatic, cluster randomised waitlist-controlled trial on integrated school mental health intervention for adolescents.* Int J Environ Res Public Health, 2021. **21**(1): p. 2034.
28. Ogawa, S., et al., *Effectiveness of Universal School-Based Mental Health Awareness Programs Among Youth in the United States: A Systematic Review.* Early Interv Psychiatry, 2016. **86**(12): p. 922-931.
29. Conley, C.S., J.A. Durlak, and A.C. Kirsch, *A Meta-analysis of universal mental health prevention programs for higher education students.* Prev Sci, 2015. **16**(4): p. 487-507.
30. Worsley, J.D. and A. Pennington, *Supporting mental health and wellbeing of university and college students: A systematic review of review-level evidence of interventions.* 2022. **17**(7): p. e0266725.
31. Conley, C.S., J.A. Durlak, and D.A. Dickson, *An evaluative review of outcome research on universal mental health promotion and prevention programs for higher education students.* J Am Coll Health, 2013. **61**(5): p. 286-301.
32. Davies, E.B., R. Morriss, and C. Glazebrook, *Computer-delivered and web-based interventions to improve depression, anxiety, and psychological well-being of university students: a systematic review and meta-analysis.* J Med Internet Res, 2014. **16**(5): p. e130.
33. King, N., et al., *The feasibility and effectiveness of a novel online mental health literacy course in supporting university student mental health: a pilot study.* BMC Psychiatry, 2022. **22**(1): p. 515.
34. Kurki, M., et al., *Digital mental health literacy -program for the first-year medical students' wellbeing: a one group quasi-experimental study.* BMC Med Educ, 2021. **21**(1): p. 563.
35. Singh, V., A. Kumar, and S. Gupta, *Mental Health Prevention and Promotion—A Narrative Review.* Frontiers in Psychiatry, 2022. **13**.
36. Saxena, S., et al., *Resources for mental health: scarcity, inequity, and inefficiency.* Lancet, 2007. **370**(9590): p. 878-89.
37. Raviola, G., et al., *Innovative Models in Mental Health Delivery Systems: Task Sharing Care with Non-specialist Providers to Close the Mental Health Treatment Gap.* Curr Psychiatry Rep, 2019. **21**(6): p. 44.
38. Karyotaki, E., et al., *Association of Task-Shared Psychological Interventions With Depression Outcomes in Low- and Middle-Income Countries: A Systematic Review and Individual Patient Data Meta-analysis.* JAMA Psychiatry, 2022. **79**(5): p. 430-443.
39. Carter, H., et al., *The emergence of digital mental health in low-income and middle-income countries: A review of recent advances and implications for the treatment and prevention of mental disorders.* J Psychiatr Res, 2021. **133**: p. 223-246.
40. Gibbs, A., et al., *Opportunities for technologically driven dialogical health communication for participatory interventions: Perspectives from male peer navigators in rural South Africa.* Social Science & Medicine, 2022. **292**: p. 114539.
41. Rose-Clarke, K., et al., *Peer-facilitated community-based interventions for adolescent health in low- and middle-income countries: A systematic review.* PLoS One, 2019. **14**(1): p. e0210468.
42. Vanessa, C.D., et al., *Effect of support group peer facilitator training programmes on peer facilitator and support group member outcomes: a systematic review.* BMJ Open, 2016. **6**(11): p. e013325.

For peer review only

For peer review only - http://bmjopen.bmj.com/site/about/guidelines.xhtml

43. Cabassa, L.J., et al., *Peer-based health interventions for people with serious mental illness: A systematic literature review.* J Psychiatr Res, 2017. **84**: p. 80-89.
44. Chien, W.T., et al., *Peer support for people with schizophrenia or other serious mental illness.* Cochrane Database Syst Rev, 2019. **4**(4): p. Cd010880.
45. Laursen, B. and R. Veenstra, *Toward understanding the functions of peer influence: A summary and synthesis of recent empirical research.* 2021. **31**(4): p. 889-907.
46. Arksey, H. and L. O'Malley, *Scoping studies: towards a methodological framework.* International Journal of Social Research Methodology, 2005. **8**(1): p. 19-32.
47. Levac, D., H. Colquhoun, and K.K. O'Brien, *Scoping studies: advancing the methodology.* Implementation Science, 2010. **5**(1): p. 69.
48. JBI, *The Joanna Briggs Institute reviewers' manual 2015: methodology for JBI scoping reviews, 2015.* . 2015.
49. Peters, M.D.J., et al., *Best practice guidance and reporting items for the development of scoping review protocols.* JBI Evid Synth, 2022. **20**(4): p. 953-968.
50. Sterne, J.A., et al., *ROBINS-I: a tool for assessing risk of bias in non-randomised studies of interventions.* Bmj, 2016. **355**: p. i4919.
51. Kennedy, C.E., et al., *The Evidence Project risk of bias tool: assessing study rigor for both randomized and non-randomized intervention studies.* Systematic Reviews, 2019. **8**(1): p. 3.
52. Hoffmann, T.C., et al., *Better reporting of interventions: template for intervention description and replication (TIDieR) checklist and guide.* Bmj, 2014. **348**: p. g1687.
53. Tricco, A.C., et al., *PRISMA Extension for Scoping Reviews (PRISMA-ScR): Checklist and Explanation.* Ann Intern Med, 2018. **169**(7): p. 467-473.
54. United, N., *Transforming our world : the 2030 Agenda for Sustainable Development*. 2015.
55. Mahlangu, P. and M. Machisa, *Preliminary evidence of promise of a sexual violence risk reduction intervention for female students in South African tertiary education institutions.* 2022. **17**(11): p. 2720-2736.
56. Mason, H., *Stress-Management Strategies among First-Year Students at a South African University: A Qualitative Study.* Journal of Student Affairs in Africa, 2017. **5**.
57. Bantjes, J., et al., *The mental health of university students in South Africa: Results of the national student survey.* Journal of Affective Disorders, 2023. **321**: p. 217-226.
58. Langtree, E., A. Razak, and F. Haffejee, *Factors causing stress among first-year students attending a nursing college in KwaZulu-Natal, South Africa.* African Journal of Health Professions Education, 2018. **10**: p. 90.
59. Skivington, K. and L. Matthews, *A new framework for developing and evaluating complex interventions: update of Medical Research Council guidance.* 2021. **374**: p. n2061.
60. Movsisyan, A., et al., *Adapting evidence-informed complex population health interventions for new contexts: a systematic review of guidance.* Implement Sci, 2019. **14**(1): p. 105.
61. Machisa, M.T. and E. Chirwa, *Suicidal Thoughts, Depression, Post-Traumatic Stress, and Harmful Alcohol Use Associated with Intimate Partner Violence and Rape Exposures among Female Students in South Africa.* 2022. **19**(13).
62. Rebecca, F.-M., et al., *Experiences across the spectrum of sexual violence are associated with decreased mental health for female university students in Eswatini.* medRxiv, 2022: p. 2022.09.19.22280102.

For peer review only

For peer review only - http://bmjopen.bmj.com/site/about/guidelines.xhtml

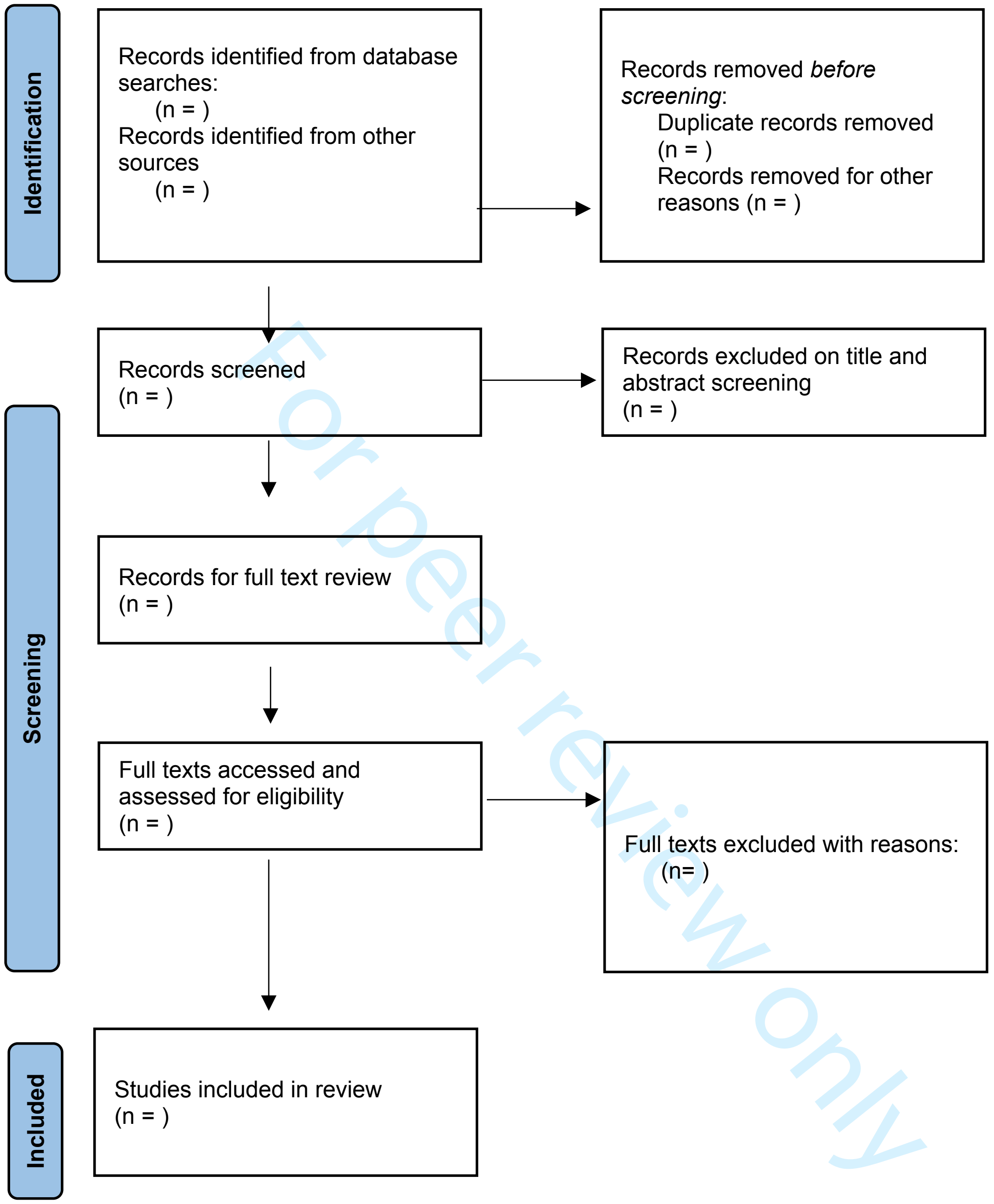

Figure 1 PRISMA Flow diagram describing the process of article review and inclusion and exclusion for the scoping review.

ID	Author	Publication date	Country/ Countries	Aims	Population and sample size	Methods

For peer review only

Intervention type, comparisons	Duration of intervention	Outcomes and details of measures	Other comments of relevance for intervention development

For peer review only

BMJ Open

Scoping review protocol for group format, peer-facilitated mental health promotion interventions for students in higher education settings

Journal:	*BMJ Open*
Manuscript ID	bmjopen-2023-080629.R1
Article Type:	Protocol
Date Submitted by the Author:	18-Mar-2024

Complete List of Authors:	Brooke-Sumner, Carrie; South African Medical Research Council, Mental Health, Alcohol, Substance Use and Tobacco Research Unit; University of KwaZulu Natal, School of Nursing and Public Health, College of Health Sciences, Howard College Campus<br>Machisa, Mercilene T.; South African Med Res Council, Gender and Health Research Unit; University of KwaZulu-Natal, South Africa, School of Nursing and Public Health, Howard College<br>Sikweyiya, Yandisa; South African Medical Research Council, Gender & Health Research Unit; University of the Witwatersrand, School of Public Health<br>Mahlangu , Pinky; South African Medical Research Council, Gender and Health Research Unit; University of KwaZulu Natal, School of Nursing and Public Health, College of Health Sciences, Howard College Campus

**Primary Subject Heading**:	Mental health
Secondary Subject Heading:	Public health
Keywords:	MENTAL HEALTH, PUBLIC HEALTH, PSYCHIATRY

SCHOLARONE™
Manuscripts

Scoping review protocol for group format, peer-facilitated mental health promotion interventions for students in higher education settings

Authors

Carrie Brooke-Sumner[1,4], Mercy Machisa[2,3,4], Yandisa Sikweyiya[2,3,4], Pinky Mahlangu[2,3,4,5]

Affiliations

[1]Mental Health, Alcohol, Substance Use and Tobacco Research Unit, South African Medical Research Council, Francie Van Zijl Drive, Parow Valley, Cape Town 7501, South Africa

[2]Gender and Health Research Unit, South African Medical Research Council, 1 Soutpansberg Road, Pretoria, Gauteng, South Africa

[3]School of Public Health, Faculty of Health Sciences, University of Witwatersrand, Johannesburg, South Africa, 60 York Road, Parktown, Johannesburg 2193, South Africa.

[4] School of Nursing and Public Health, College of Health Sciences, Howard College Campus, University of KwaZulu-Natal, South Africa

[5] School of Health Systems and Public Health, Faculty of Health Sciences, University of Pretoria, South Africa

Corresponding author: carrie.brooke-sumner@mrc.ac.za

Word count: 3340

Abstract

Introduction

Young people in higher education face various stressors that can make them vulnerable to mental ill health. Mental health promotion in this group therefore has important potential benefits. Peer-facilitated and group format interventions may be feasible and sustainable. The scoping review outlined in this protocol aims to map the literature on group format, peer-facilitated, in-person interventions for mental health promotion for higher education students attending courses on campuses in high and low- and middle-income countries.

Methods and analysis

Relevant studies will be identified through conducting searches of electronic databases, including Medline, CINAHL, Scopus, ERIC and PsychInfo. Searches will be conducted using Boolean operators (AND, OR, NOT) and truncation functions appropriate for each database. We will include a grey literature search. We will include articles from student participants of any gender,

For peer review only - http://bmjopen.bmj.com/site/about/guidelines.xhtml

and published in peer reviewed journals between 2008 and 2023. We will include English language studies and all study types including RCTs, pilot studies and descriptive studies of intervention development. A draft charting table has been developed, which includes the fields: author, publication date, country/countries, aims, population and sample size, demographics, methods, intervention type, comparisons, peer training, number of sessions/duration of intervention, outcomes and details of measures.

Ethics and dissemination

No primary data will be collected from research participants to produce this review so ethics committee approval is not required. All data will be collated from published peer reviewed studies already in the public domain. We will publish the review in an open access, peer reviewed journal accessible to researchers in low- and middle-income countries. This protocol is registered on Open Science Framework (https://osf.io/agbfj/).

Article summary

Strengths and limitations

- This scoping review will map literature on group format, peer-facilitated and in-person interventions for mental health promotion among higher education students globally.
- The methods are grounded in established methods and guidance for scoping reviews (PRISMA).
- The review will include a search of published and grey literature to synthesize the range of interventions for group-based, in-person peer-facilitated mental health promotion for students.
- The protocol is limited in focusing on English language articles.

Keywords

Mental health, young adult, college, university, peer, mental health promotion

Background

Mental health of young people is a global public health priority [1], with COVID-19 having accentuated the urgency of addressing this area [2, 3]. Mental well-being is crucial for higher education (university/college) students to be resilient in facing the demands of academic life [4]

For peer review only - http://bmjopen.bmj.com/site/about/guidelines.xhtml

as well as being a building block for every society through youth development. Young people entering higher education face a period of substantial change, with new and varied stressors and changing interpersonal relationships, financial circumstances, and life challenges [5-7]. Academic and other stress is associated with the development of mental health conditions [5, 7]. Mental distress is thus prevalent in higher education institutions with young women students particularly vulnerable to conditions such as anxiety, depression, and post-traumatic stress disorder (e.g. [8-11]). The stressors experienced in student life overlay other vulnerability factors and social determinants [12] for developing a mental health condition, including previous experiences of childhood adversity and trauma [13, 14], and being from a sexual minority group or gender non-conforming [13]. In low- and middle-income countries (LMIC) in which human resources for mental health treatments are especially scarce [15], the potential gains from prevention of mental disorders for young people are significant and may correspond to population level benefits in the long term [16, 17].

Mental health promotion (encompassing building wellbeing and prevention of development of mental health conditions) remains underprioritized in many settings in comparison with efforts for treatment provision. However, such work is a crucial component of the World Health Organisation (WHO) mix of mental health services (which includes self-care and informal care, primary health care and specialist health care) [18]. Good mental health for young people may be contextually nuanced, but is dependent on a range of domains, including mental health literacy, attitudes towards mental health conditions, self-perceptions, cognitive skills, academic performance, emotions, behaviours, self-management, social skills, quality of significant relationships, physical and sexual health, meaning in life and quality of life [19, 20]. With this range of domains, transdiagnositic or common elements treatment and prevention approaches are relevant [17], underpinned conceptually by the building of adaptive social and emotional skills, stress management, positive self-perceptions and supportive relationships. Prevention in relation to mental ill-health also encompasses other health behaviours (e.g. dietary health, physical activity, sleep) that can have an impact on mental health outcomes [21]. Harnessing common practice elements can enable an evidence-informed approach to prevention even when a specific evidence-based intervention may be lacking (as may be the case in many LMIC settings where this research area is less developed) [17, 22].

For peer review only - http://bmjopen.bmj.com/site/about/guidelines.xhtml

Given the breadth of domains of good mental health for higher education students, mental health promotion and prevention interventions necessarily cover a wide variety of interventions at the individual, community and societal levels [23] which can be universal (population based) or selective (targeting individuals or subgroups at higher risk) [16, 24]. Young people in higher education settings present a particular set of opportunities for preventive and promotive interventions given the social connectedness of student populations. Many mental health conditions have their onset in adolescence. This is a key point for intervention and although not the focus of this review, there is developing evidence that school-based prevention can be effective in improving mental health literacy and reducing mental health stigma [25-28].

There is also a developing systematized evidence base for higher education student mental health promotion from high income countries (HIC). Systematic reviews of prevention programmes for student mental health showed moderate effects for common practice elements including psychoeducation (mental health literacy training), relaxation techniques, and cognitive restructuring [19]. Guided mental health skills training programmes [22, 29-31] and computer and web-based interventions delivered by a variety of professionals [32-34] similarly showed moderate effects. Current developments in mental health promotion include a movement towards a positive conceptualisation of mental health literacy which is not focused on 'illness' and symptoms. Within this conceptualisation, positive mental health literacy includes problem solving ability, independence, relational skills and self-control [35]. A similar evidence base for student mental health promotion in LMIC is required [36] building on evidence for approaches from HIC, but with consideration for contextual differences in relation to availability of human and other resources.

Most notably, a key aspect of mental health prevention interventions is the delivery agent. In LMIC settings specialists such as psychologists, social workers and counsellors are scarce [37] and task-sharing (service provision by non-specialists with upward referral if necessary) has long been advocated as a feasible approach for mental health service delivery [23, 38, 39]. Digital interventions have also been put forward as a low-resource approach for mental health promotion [36]. Evidence for digital interventions for mental health and access to technology in HIC and

For peer review only - http://bmjopen.bmj.com/site/about/guidelines.xhtml

LMIC is growing and indicates potential for these approaches, in conjunction with other supportive service options [40, 41]. In-person interventions enable building of rapport and support and are needed in contexts where access to computer or mobile technology may be limited. Group approaches may be a feasible low-resource approach in some LMIC contexts. Hence in-person group approaches are the focus of this review, which will inform development of in-person group interventions. The group format presents the opportunity for reaching higher numbers of students in a less resource intensive approach than individual level support, which may be advantageous for mental health promotion [36]. Further to this, peer-led approaches, in which people who share a common lived experience (e.g. being a higher education student) are involved in providing services, have traction in other areas of public health, for young people in particular (e.g. [42, 43]) and in other areas of mental health service provision (e.g. [44, 45]). Peer-facilitated approaches may be particularly appropriate for mental health promotion amongst young people given the importance of peer influence at this point in the life course [46]. These approaches have the potential to be low resource and sustainable in resource constrained settings [42], though still requiring investment and engagement [47] to be productively implemented.

This scoping review will map the evidence for in-person, group-based, peer-facilitated mental health promotion interventions for young people in higher education settings globally. Once conducted, this review will enable progress in bringing together evidence for such preventive approaches that are applicable for resource-constrained LMIC contexts. This will serve as a step towards building the field of evidence-informed mental health promotion among students in higher education settings in LMIC.

Research questions

1. What range of in-person, group-based, peer-facilitated mental health promotion interventions are offered to higher education students on campuses globally?
2. What are the common practices, processes and outcome measures used for these interventions?

Objectives

1. To map the literature on group-based, peer-facilitated, in-person group interventions for mental health promotion for higher education students on campuses globally.

For peer review only - http://bmjopen.bmj.com/site/about/guidelines.xhtml

2. To describe these peer-facilitated group interventions and the ways they have been evaluated.
3. To describe common practice elements and those identified as having positive effects, no effects, or negative effects, if possible.

Methods and analysis

Methodology for the review will be based on that presented by Arksey and O'Malley [48] and further developed by Levac [49]. A key recommendation from this advancement relates to clarifying and linking the purpose of the review with the research question [49]. The purpose of the review will be to identify common practice elements of group-format, peer-facilitated mental health promotion interventions used in higher education populations. We will include a grey literature search as recommended by Joanna Briggs Institute Guidance [50, 51]. We will also use an iterative approach for identifying and selecting studies [49] including identifying studies from reference lists of relevant studies with discussion of the review team. The following steps will be undertaken to investigate the review question and are detailed in the sections following:

1. Identifying the research question
2. Identifying relevant studies
3. Study selection
4. Charting the data
5. Collating, summarizing, and reporting results

1.Identifying the research question

The Population Concept Context (PCC) mnemonic has been identified as appropriate for scoping review methodology [50]. The population of interest is defined as students studying at higher education campuses; the concept is defined as any mental health promotion intervention delivered in-person by a peer and in a group format; context is defined as articles from HIC and LMIC. This scoping review therefore has the following research questions:

Primary research question

- What evidence is available globally for peer-facilitated, group-format in-person interventions for mental health promotion for students on higher education campuses?

Secondary research questions

For peer review only - http://bmjopen.bmj.com/site/about/guidelines.xhtml

- What are the common practice elements and delivery methods for interventions reported in this body of literature?
- What research gaps are identified by the literature included in the scoping review?

2. Identifying relevant studies

The review will be conducted from June to December 2024. Relevant studies will be identified through conducting searches of electronic databases, including Medline, CINAHL, Scopus, PsychInfo and ERIC. Searches will be conducted using Boolean operators (AND, OR, NOT) and truncation functions appropriate for each database. Search strings will be developed in line with literature on peer approaches in higher education [52] and according to each database. Searches will include for example: "mental health literacy" OR "mental health promotion" OR "psychoeducation" AND "intervention" OR "program" OR "mental health intervention" AND "group intervention" OR "peer" OR "peer support" OR "peer health education" AND "student" OR "campus" OR "university" OR "college" AND "depression" OR "anxiety" OR "mental health literacy" OR "positive mental health" OR "self care" OR "resilience" or "stress management". We will seek assistance from a specialist librarian in refining search strings for databases based on these key terms to generate search results feasible for review by the study team. We will take an iterative approach to the searches [50] to include relevant keywords and to maintain feasibility of the number of records to review. Review authors will conduct backward citation tracking for all articles selected for inclusion as an approach to identify further relevant studies. If required, review authors will contact corresponding authors of these articles to identify relevant studies [50]. Relevant studies will be downloaded and managed using Raayan to conduct screening. The grey literature search will be conducted with terms above using databases such as WorldCat, OpenMD, Eldis, OpenGrey, Canadian Agency for Drugs and Technologies in Health (CADTH) and Grey Matters. Programme reports, case studies and manuals will be included.

3. Study selection

Titles and abstracts of identified studies will be subjected to double screening for relevance, with records divided among authors to facilitate faster screening. Titles and abstracts will be screened according to the pre-developed inclusion and exclusion criteria developed for answering the review question (Figure 1). Articles identified as relevant in this first screening process will then be included in a full text review conducted by all authors (double screening, two authors review

For peer review only - http://bmjopen.bmj.com/site/about/guidelines.xhtml

each article). Agreement and disagreement on full text inclusion will be discussed by reviewing authors, and any disagreements resolved through discussion with the full review team.

Inclusion criteria

The purposes of the scoping review mean that included articles will describe in-person interventions delivered on higher education campuses in HIC and LMIC, by peers. Articles from HIC will be included as there may be practice elements and delivery approaches from HIC that are relevant for advancing the field in LMIC where peer-delivered approaches may be less well-developed. We will include articles that describe group-format interventions delivered in higher education settings among student participants of any gender, and published in peer reviewed journals between 2008 and 2024 (15 year range for feasibility in number of records to review, and to capture recent developments in the field). We will include English language studies and all study types including RCTs, pilot studies and descriptive studies of intervention development. We will not exclude papers that do not report where the study was conducted.

Exclusion criteria

Articles describing individual and group interventions delivered by clinical professionals (e.g. psychologists, social workers, counsellors or other university staff) will be excluded, as well as articles reporting on clinical treatment interventions as opposed to prevention interventions. Articles reporting on interventions not delivered in-person, by peers or in groups (e.g. online/web based) will also be excluded. Non-English language papers and those published outside of the time period indicated will be excluded.

4. Data extraction and management

Data extraction will be conducted from PDF or other electronic files into an Excel-based data management file (Supplementary File 1). We will conduct a pilot test of this abstraction form with initial articles to ensure consistency in data extraction between team members.

5. Charting the data

The charting process will provide a descriptive summary of the evidence presented in included studies [50], as the initial step towards answering the primary and secondary research questions. The draft charting table includes the fields: author, publication date, country/countries, aims, population and sample size, methods, demographics of peers and/or target population, intervention type, peer training, number of sessions/duration, retention, comparisons, duration of intervention,

For peer review only - http://bmjopen.bmj.com/site/about/guidelines.xhtml

outcomes and details of measures. The data charting table completed for each reviewer will be collated and reviewed by the whole review team to address any inconsistencies.

6. Assessment of study quality

For this scoping review we will not conduct a formal assessment of Risk of Bias using one of the established reporting tools (e.g. [53, 54]) as the aim is to provide an overview of existing evidence regardless of methodological quality. We will however use The Template for Intervention Description and Replication (TIDieR) [55] checklist to assess completeness in the reporting of included intervention studies. Available since 2014 this guide may have been used by study authors to describe interventions, and can be used in this scoping review to provide a characterization of interventions along with charting of data.

7. Summarizing and reporting findings

Appropriate tables will be used to give the reader an overall picture of the spread of evidence across publication years and country of origin. A narrative description of the studies will also be presented (including TIDieR), aligning findings with the primary and secondary research questions [50]. For ease of use for the reader, findings will be presented under the conceptual headings “Definition of peer”, “Intervention practice elements”, “Intervention delivery” and “Research gaps”. The final manuscript will be prepared using the PRISMA extension for scoping reviews [56]. Depending on the amount of literature found, data abstraction may also include categorizing the extracted information into themes to identify patterns across the literature, and according to high income, low-income or lower-middle income country status (given differences in availability of resources across these settings).

Ethical considerations and Dissemination

No primary data will be collected from research participants to produce this review. All data will be collated from published peer reviewed studies already in the public domain. This study therefore does not require ethical approval from a Human Research Ethics Committee. To minimize potential bias and ensure an accurate reflection of the scope of the literature we have a review team with four members who will work collaboratively. These researchers are senior scientists at the South African Medical Research Council, with backgrounds in quantitative and qualitative

For peer review only - http://bmjopen.bmj.com/site/about/guidelines.xhtml

research in public health and mental health. We will publish the review in an open access, peer reviewed journal accessible to researchers in LMIC. We will also disseminate findings through our established Community Advisory Board and stakeholder engagement platforms of the South African Medical Research Council.

Patient and public involvement Patients and/or the public were not involved in the design, or conduct, or reporting, or dissemination plans of this research.

Discussion

The United Nations Sustainable Development Goals (SDGs) prioritise mental wellness in SDG 3 [57]. This indicates high level commitment to improvement in mental wellbeing, which requires a response from all sectors of society, and quality research to guide this response [58]. Addressing social determinants of mental health [12], the social context that precedes the development of mental health conditions (e.g. through mental health promotion), holds great potential for prevention of the growing burden of mental disorders globally and in young people in particular who make up a large proportion of the global population. Young people in higher education in particular face developmental and academic stressors which can make them vulnerable to mental health conditions [59-61].

Prevention efforts that promote mental health will need to begin to address vulnerabilities as well as improve coping and resilience. Intervention research to develop and test interventions in this area is required. [58]. The scoping review outlined in this protocol will contribute to moving the mental health promotion field in LMIC forward through mapping group-format, peer-facilitated mental health promotion practice elements. This will add to the body of work on adaptation of complex interventions [62, 63], incorporating learnings from HIC settings with practice elements that are appropriate and can be tailored for LMIC settings. Evidence for approaches that are feasible, acceptable, and crucially - tailored - for LMIC contexts is required, given the risk factors associated with mental health conditions in these settings [60, 64]. For example such approaches may give prominence to developing social support, promoting aspects of wellbeing including social, academic, and spiritual wellbeing, and building resilience and coping strategies which are crucial in LMIC contexts [65]. Through mapping the body of literature this scoping review will also contribute to evidence for state of the field of task-sharing for mental health promotion in

For peer review only - http://bmjopen.bmj.com/site/about/guidelines.xhtml

LMIC settings. Finally, findings from the review will identify gaps and opportunities for researchers and practitioners interested in implementing group-format, peer-led approaches for improving mental health for students in LMIC and in HIC.

Funding statement

CBS, MM, PM and YS are supported in this work by funds from the South African Medical Research Council Flagship projects funding.

Author contributions

All authors conceptualized the study and developed the methods collaboratively. PM, MM and YS contributed to conceptualization of research questions, development of search strategy and scoping review methods. CBS led the development of the research questions and methods and produced the initial draft of this manuscript, all authors reviewed and approved the final manuscript.

Competing interests

None declared.

Patient consent for publication

Not applicable.

Provenance and peer review

Not commissioned; externally peer reviewed.

Data availability statement

All data for the review will be made available on reasonable request from Kholiswa Dube Kholiswa.Dube@mrc.ac.za or Carrie Brooke-Sumner carrie.brooke-sumner@mrc.ac.za.

Supplemental material

Data charting template.

Figure 1 PRISMA Flow diagram describing the process of article review and inclusion and exclusion for the scoping review.

References

1. Patel, V., et al., *Mental health of young people: a global public-health challenge.* Lancet, 2007. **369**(9569): p. 1302-1313.
2. WHO, *Mental Health and COVID-19: Early evidence of the pandemic's impact*. 2022, World Health Organization: Geneva.

For peer review only

For peer review only - http://bmjopen.bmj.com/site/about/guidelines.xhtml

3. Visser, M. and E. Law-van Wyk, *University students' mental health and emotional wellbeing during the COVID-19 pandemic and ensuing lockdown.* South African Journal of Psychology, 2021. **51**(2): p. 229-243.

4. Ahorsu, D.K., et al., *Effect of a peer-led intervention combining mental health promotion with coping-strategy-based workshops on mental health awareness, help-seeking behavior, and wellbeing among university students in Hong Kong.* International Journal of Mental Health Systems, 2021. **15**(1): p. 1-10.

5. Karyotaki, E., et al., *Sources of Stress and Their Associations With Mental Disorders Among College Students: Results of the World Health Organization World Mental Health Surveys International College Student Initiative.* Front Psychol, 2020. **11**: p. 1759.

6. Jessop, D.C., C. Herberts, and L. Solomon, *The impact of financial circumstances on student health.* Br J Health Psychol, 2005. **10**(Pt 3): p. 421-39.

7. Liu, X., S. Ping, and W. Gao, *Changes in Undergraduate Students' Psychological Well-Being as They Experience University Life.* Int J Environ Res Public Health, 2019. **16**(16).

8. Macaskill, A., *The mental health of university students in the United Kingdom.* British Journal of Guidance & Counselling, 2013. **41**(4): p. 426-441.

9. Storrie, K., K. Ahern, and A. Tuckett, *A systematic review: Students with mental health problems--a growing problem.* Int J Nurs Pract, 2010. **16**(1): p. 1-6.

10. Rotenstein, L.S., et al., *Prevalence of Depression, Depressive Symptoms, and Suicidal Ideation Among Medical Students: A Systematic Review and Meta-Analysis.* Jama, 2016. **316**(21): p. 2214-2236.

11. Eskin, M., et al., *Suicidal Behavior and Psychological Distress in University Students: A 12-nation Study.* Arch Suicide Res, 2016. **20**(3): p. 369-88.

12. Lund, C., et al., *Social determinants of mental disorders and the Sustainable Development Goals: a systematic review of reviews.* Lancet Psychiatry, 2018. **5**(4): p. 357-369.

13. Campbell, F., et al., *Factors that influence mental health of university and college students in the UK: a systematic review.* BMC Public Health, 2022. **22**(1): p. 1-22.

14. Berenz, E.C., et al., *Posttraumatic stress disorder symptoms, anxiety sensitivity, and alcohol-use motives in college students with a history of interpersonal trauma.* Psychol Addict Behav, 2016. **30**(7): p. 755-763.

15. Rathod, S., et al., *Mental Health Service Provision in Low- and Middle-Income Countries.* Health Serv Insights, 2017. **10**: p. 1178632917694350.

16. Salazar de Pablo, G., et al., *Universal and selective interventions to promote good mental health in young people: Systematic review and meta-analysis.* Eur Neuropsychopharmacol, 2020. **41**: p. 28-39.

17. Colizzi, M., A. Lasalvia, and M. Ruggeri, *Prevention and early intervention in youth mental health: is it time for a multidisciplinary and trans-diagnostic model for care?* International Journal of Mental Health Systems, 2020. **14**(1): p. 23.

18. Shidhaye R, L.C., Chisholm D., *Health Care Platform Interventions. Mental, Neurological, and Substance Use Disorders: Disease Control Priorities*, C.D. Patel V, Dua T, et al., editors., Editor. 2016, The World Bank: Washington (DC).

19. Fusar-Poli, P., et al., *What is good mental health? A scoping review.* Eur Neuropsychopharmacol, 2020. **31**: p. 33-46.

20. Dodd, A.L. and M. Priestley, *University student well-being in the United Kingdom: a scoping review of its conceptualisation and measurement.* 2021. **30**(3): p. 375-387.

21. Hutchesson, M.J., et al., *Health behavior interventions for university students measuring mental health outcomes: A scoping review.* Front Public Health, 2022. **10**: p. 1063429.

For peer review only - http://bmjopen.bmj.com/site/about/guidelines.xhtml

22. Rith-Najarian, L.R., M.M. Boustani, and B.F. Chorpita, *A systematic review of prevention programs targeting depression, anxiety, and stress in university students.* J Affect Disord, 2019. **257**: p. 568-584.

23. Petersen, I., et al., *Promotion, prevention and protection: interventions at the population- and community-levels for mental, neurological and substance use disorders in low- and middle-income countries.* Int J Ment Health Syst, 2016. **10**: p. 30.

24. Organization, W.H., *Prevention of Mental Disorders: Effective Interventions and Policy Options: Summary Report*. 2004: Geneva.

25. Ma, K.K.Y. and J.K. Anderson, *Review: School-based interventions to improve mental health literacy and reduce mental health stigma - a systematic review.* 2023. **28**(2): p. 230-240.

26. Ma, K.K.Y. and A.M. Burn, *Review: School-based mental health literacy interventions to promote help-seeking - a systematic review.* 2022.

27. Amudhan, S., *Project SUMS (scaling up of mental health in schools): design and methods for a pragmatic, cluster randomised waitlist-controlled trial on integrated school mental health intervention for adolescents.* Int J Environ Res Public Health, 2021. **21**(1): p. 2034.

28. Ogawa, S., et al., *Effectiveness of Universal School-Based Mental Health Awareness Programs Among Youth in the United States: A Systematic Review.* Early Interv Psychiatry, 2016. **86**(12): p. 922-931.

29. Conley, C.S., J.A. Durlak, and A.C. Kirsch, *A Meta-analysis of universal mental health prevention programs for higher education students.* Prev Sci, 2015. **16**(4): p. 487-507.

30. Worsley, J.D. and A. Pennington, *Supporting mental health and wellbeing of university and college students: A systematic review of review-level evidence of interventions.* 2022. **17**(7): p. e0266725.

31. Conley, C.S., J.A. Durlak, and D.A. Dickson, *An evaluative review of outcome research on universal mental health promotion and prevention programs for higher education students.* J Am Coll Health, 2013. **61**(5): p. 286-301.

32. Davies, E.B., R. Morriss, and C. Glazebrook, *Computer-delivered and web-based interventions to improve depression, anxiety, and psychological well-being of university students: a systematic review and meta-analysis.* J Med Internet Res, 2014. **16**(5): p. e130.

33. King, N., et al., *The feasibility and effectiveness of a novel online mental health literacy course in supporting university student mental health: a pilot study.* BMC Psychiatry, 2022. **22**(1): p. 515.

34. Kurki, M., et al., *Digital mental health literacy -program for the first-year medical students' wellbeing: a one group quasi-experimental study.* BMC Med Educ, 2021. **21**(1): p. 563.

35. Carvalho, D., et al., *Positive Mental Health Literacy: A Concept Analysis.* Front Psychol, 2022. **13**: p. 877611.

36. Singh, V., A. Kumar, and S. Gupta, *Mental Health Prevention and Promotion—A Narrative Review.* Frontiers in Psychiatry, 2022. **13**.

37. Saxena, S., et al., *Resources for mental health: scarcity, inequity, and inefficiency.* Lancet, 2007. **370**(9590): p. 878-89.

38. Raviola, G., et al., *Innovative Models in Mental Health Delivery Systems: Task Sharing Care with Non-specialist Providers to Close the Mental Health Treatment Gap.* Curr Psychiatry Rep, 2019. **21**(6): p. 44.

39. Karyotaki, E., et al., *Association of Task-Shared Psychological Interventions With Depression Outcomes in Low- and Middle-Income Countries: A Systematic Review and Individual Patient Data Meta-analysis.* JAMA Psychiatry, 2022. **79**(5): p. 430-443.

40. Carter, H., et al., *The emergence of digital mental health in low-income and middle-income countries: A review of recent advances and implications for the treatment and prevention of mental disorders.* J Psychiatr Res, 2021. **133**: p. 223-246.

For peer review only

For peer review only - http://bmjopen.bmj.com/site/about/guidelines.xhtml

41. Gibbs, A., et al., *Opportunities for technologically driven dialogical health communication for participatory interventions: Perspectives from male peer navigators in rural South Africa.* Social Science & Medicine, 2022. **292**: p. 114539.
42. Rose-Clarke, K., et al., *Peer-facilitated community-based interventions for adolescent health in low- and middle-income countries: A systematic review.* PLoS One, 2019. **14**(1): p. e0210468.
43. Vanessa, C.D., et al., *Effect of support group peer facilitator training programmes on peer facilitator and support group member outcomes: a systematic review.* BMJ Open, 2016. **6**(11): p. e013325.
44. Cabassa, L.J., et al., *Peer-based health interventions for people with serious mental illness: A systematic literature review.* J Psychiatr Res, 2017. **84**: p. 80-89.
45. Chien, W.T., et al., *Peer support for people with schizophrenia or other serious mental illness.* Cochrane Database Syst Rev, 2019. **4**(4): p. Cd010880.
46. Laursen, B. and R. Veenstra, *Toward understanding the functions of peer influence: A summary and synthesis of recent empirical research.* 2021. **31**(4): p. 889-907.
47. Haas, J., Byrom, N., Foster, J. et al., *Staff Perspectives: Defining the Types, Challenges and Lessons Learnt of University Peer Support for Student Mental Health and Wellbeing.* Education Sciences, 2023. **13**(9).
48. Arksey, H. and L. O'Malley, *Scoping studies: towards a methodological framework.* International Journal of Social Research Methodology, 2005. **8**(1): p. 19-32.
49. Levac, D., H. Colquhoun, and K.K. O'Brien, *Scoping studies: advancing the methodology.* Implementation Science, 2010. **5**(1): p. 69.
50. JBI, *The Joanna Briggs Institute reviewers' manual 2015: methodology for JBI scoping reviews, 2015.* . 2015.
51. Peters, M.D.J., et al., *Best practice guidance and reporting items for the development of scoping review protocols.* JBI Evid Synth, 2022. **20**(4): p. 953-968.
52. Pointon-Haas, J., et al., *A systematic review of peer support interventions for student mental health and well-being in higher education.* BJPsych Open, 2023. **10**(1): p. e12.
53. Sterne, J.A., et al., *ROBINS-I: a tool for assessing risk of bias in non-randomised studies of interventions.* Bmj, 2016. **355**: p. i4919.
54. Kennedy, C.E., et al., *The Evidence Project risk of bias tool: assessing study rigor for both randomized and non-randomized intervention studies.* Systematic Reviews, 2019. **8**(1): p. 3.
55. Hoffmann, T.C., et al., *Better reporting of interventions: template for intervention description and replication (TIDieR) checklist and guide.* Bmj, 2014. **348**: p. g1687.
56. Tricco, A.C., et al., *PRISMA Extension for Scoping Reviews (PRISMA-ScR): Checklist and Explanation.* Ann Intern Med, 2018. **169**(7): p. 467-473.
57. United, N., *Transforming our world : the 2030 Agenda for Sustainable Development*. 2015.
58. Mahlangu, P. and M. Machisa, *Preliminary evidence of promise of a sexual violence risk reduction intervention for female students in South African tertiary education institutions.* 2022. **17**(11): p. 2720-2736.
59. Mason, H., *Stress-Management Strategies among First-Year Students at a South African University: A Qualitative Study.* Journal of Student Affairs in Africa, 2017. **5**.
60. Bantjes, J., et al., *The mental health of university students in South Africa: Results of the national student survey.* Journal of Affective Disorders, 2023. **321**: p. 217-226.
61. Langtree, E., A. Razak, and F. Haffejee, *Factors causing stress among first-year students attending a nursing college in KwaZulu-Natal, South Africa.* African Journal of Health Professions Education, 2018. **10**: p. 90.
62. Skivington, K. and L. Matthews, *A new framework for developing and evaluating complex interventions: update of Medical Research Council guidance.* 2021. **374**: p. n2061.

For peer review only - http://bmjopen.bmj.com/site/about/guidelines.xhtml

63. Movsisyan, A., et al., *Adapting evidence-informed complex population health interventions for new contexts: a systematic review of guidance.* Implement Sci, 2019. **14**(1): p. 105.
64. Machisa, M.T. and E. Chirwa, *Suicidal Thoughts, Depression, Post-Traumatic Stress, and Harmful Alcohol Use Associated with Intimate Partner Violence and Rape Exposures among Female Students in South Africa.* 2022. **19**(13).
65. Rebecca, F.-M., et al., *Experiences across the spectrum of sexual violence are associated with decreased mental health for female university students in Eswatini.* medRxiv, 2022: p. 2022.09.19.22280102.

For peer review only

For peer review only - http://bmjopen.bmj.com/site/about/guidelines.xhtml

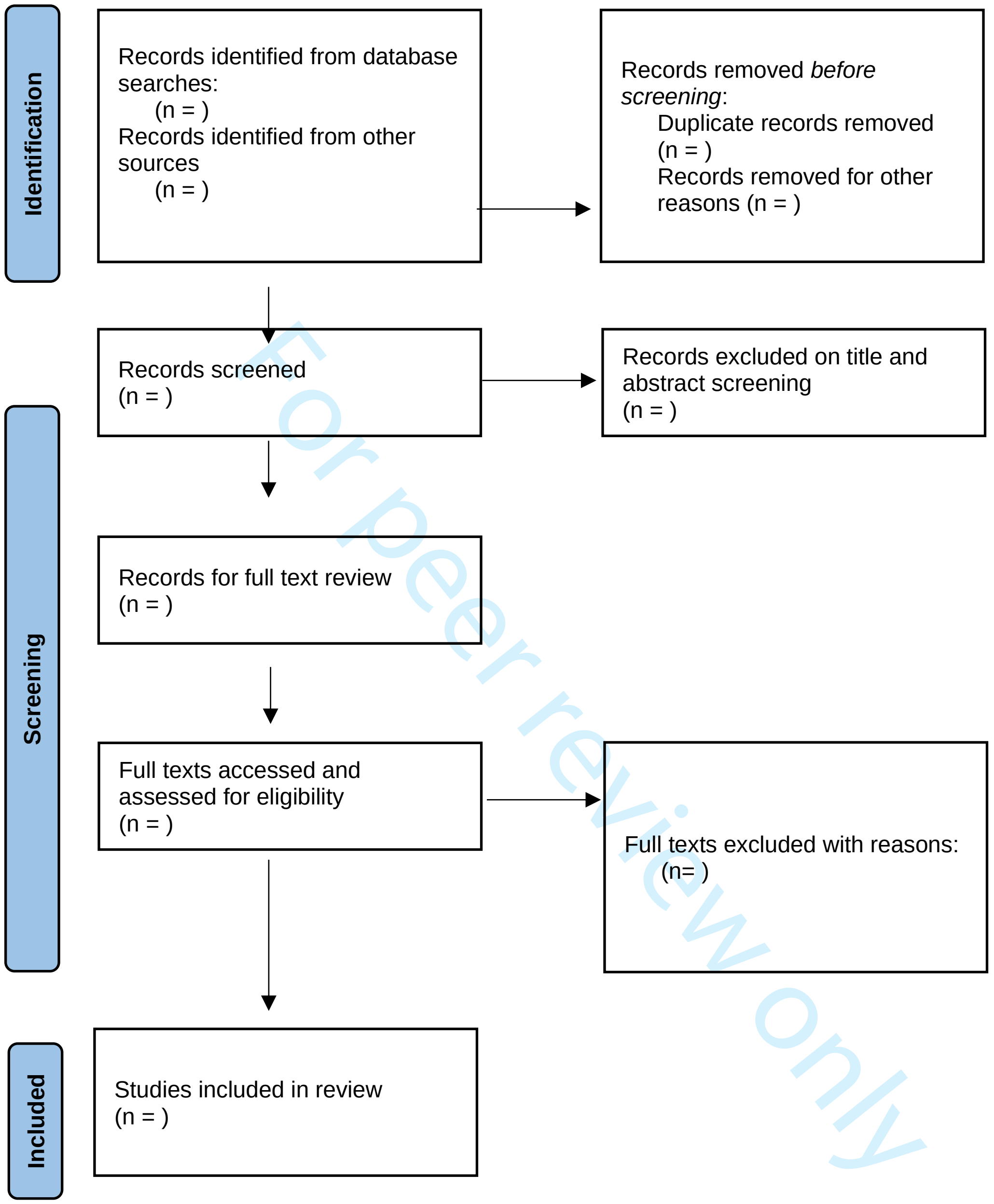

Figure 1 PRISMA Flow diagram describing the process of article review and inclusion and exclusion for the scoping review.

ID	Author	Publication date	Country	Aims	Population and sample size	Demographics of peers/target population	Methods	Intervention type, comparisons	Peer training	Number of sessions/ Duration of intervention	Outcomes and details of measures	Other comments of relevance for intervention development

For peer review only

Preferred Reporting Items for Systematic reviews and Meta-Analyses extension for Scoping Reviews (PRISMA-ScR) Checklist

SECTION	ITEM	PRISMA-ScR CHECKLIST ITEM	REPORTED ON PAGE #
**TITLE**			
Title	1	Identify the report as a scoping review.	
**ABSTRACT**			
Structured summary	2	Provide a structured summary that includes (as applicable): background, objectives, eligibility criteria, sources of evidence, charting methods, results, and conclusions that relate to the review questions and objectives.	
**INTRODUCTION**			
Rationale	3	Describe the rationale for the review in the context of what is already known. Explain why the review questions/objectives lend themselves to a scoping review approach.	
Objectives	4	Provide an explicit statement of the questions and objectives being addressed with reference to their key elements (e.g., population or participants, concepts, and context) or other relevant key elements used to conceptualize the review questions and/or objectives.	
**METHODS**			
Protocol and registration	5	Indicate whether a review protocol exists; state if and where it can be accessed (e.g., a Web address); and if available, provide registration information, including the registration number.	
Eligibility criteria	6	Specify characteristics of the sources of evidence used as eligibility criteria (e.g., years considered, language, and publication status), and provide a rationale.	
Information sources*	7	Describe all information sources in the search (e.g., databases with dates of coverage and contact with authors to identify additional sources), as well as the date the most recent search was executed.	
Search	8	Present the full electronic search strategy for at least 1 database, including any limits used, such that it could be repeated.	
Selection of sources of evidence†	9	State the process for selecting sources of evidence (i.e., screening and eligibility) included in the scoping review.	
Data charting process‡	10	Describe the methods of charting data from the included sources of evidence (e.g., calibrated forms or forms that have been tested by the team before their use, and whether data charting was done independently or in duplicate) and any processes for obtaining and confirming data from investigators.	
Data items	11	List and define all variables for which data were sought and any assumptions and simplifications made.	
Critical appraisal of individual sources of evidence§	12	If done, provide a rationale for conducting a critical appraisal of included sources of evidence; describe the methods used and how this information was used in any data synthesis (if appropriate).	
Synthesis of results	13	Describe the methods of handling and summarizing the data that were charted.	

For peer review only - http://bmjopen.bmj.com/site/about/guidelines.xhtml

SECTION	ITEM	PRISMA-ScR CHECKLIST ITEM	REPORTED ON PAGE #
**RESULTS**			
Selection of sources of evidence	14	Give numbers of sources of evidence screened, assessed for eligibility, and included in the review, with reasons for exclusions at each stage, ideally using a flow diagram.	
Characteristics of sources of evidence	15	For each source of evidence, present characteristics for which data were charted and provide the citations.	
Critical appraisal within sources of evidence	16	If done, present data on critical appraisal of included sources of evidence (see item 12).	
Results of individual sources of evidence	17	For each included source of evidence, present the relevant data that were charted that relate to the review questions and objectives.	
Synthesis of results	18	Summarize and/or present the charting results as they relate to the review questions and objectives.	
**DISCUSSION**			
Summary of evidence	19	Summarize the main results (including an overview of concepts, themes, and types of evidence available), link to the review questions and objectives, and consider the relevance to key groups.	
Limitations	20	Discuss the limitations of the scoping review process.	
Conclusions	21	Provide a general interpretation of the results with respect to the review questions and objectives, as well as potential implications and/or next steps.	
**FUNDING**			
Funding	22	Describe sources of funding for the included sources of evidence, as well as sources of funding for the scoping review. Describe the role of the funders of the scoping review.	

JBI = Joanna Briggs Institute; PRISMA-ScR = Preferred Reporting Items for Systematic reviews and Meta-Analyses extension for Scoping Reviews.

* Where *sources of evidence* (see second footnote) are compiled from, such as bibliographic databases, social media platforms, and Web sites.

† A more inclusive/heterogeneous term used to account for the different types of evidence or data sources (e.g., quantitative and/or qualitative research, expert opinion, and policy documents) that may be eligible in a scoping review as opposed to only studies. This is not to be confused with *information sources* (see first footnote).

‡ The frameworks by Arksey and O'Malley (6) and Levac and colleagues (7) and the JBI guidance (4, 5) refer to the process of data extraction in a scoping review as data charting.

§ The process of systematically examining research evidence to assess its validity, results, and relevance before using it to inform a decision. This term is used for items 12 and 19 instead of "risk of bias" (which is more applicable to systematic reviews of interventions) to include and acknowledge the various sources of evidence that may be used in a scoping review (e.g., quantitative and/or qualitative research, expert opinion, and policy document).

From: Tricco AC, Lillie E, Zarin W, O'Brien KK, Colquhoun H, Levac D, et al. PRISMA Extension for Scoping Reviews (PRISMAScR): Checklist and Explanation. Ann Intern Med. 2018;169:467–473. doi: 10.7326/M18-0850.

For peer review only - http://bmjopen.bmj.com/site/about/guidelines.xhtml
